# Pathway analysis for genetic association studies: to do, or not to do? That is the question

**DOI:** 10.1186/1753-6561-8-S1-S103

**Published:** 2014-06-17

**Authors:** Line Dufresne, Karim Oualkacha, Vincenzo Forgetta, Celia MT Greenwood

**Affiliations:** 1 Department of Epidemiology, Biostatistics and Occupational Health, McGill University, 1020 Pine Avenue West, Montreal, Quebec, H3A 1A2, Canada; 2 Département de Mathématiques, Université du Québec à Montréal, PK-5151, 201 avenue du Président-Kennedy, Montréal, QC H2X 3Y7, Canada; 3 Lady Davis Institute for Medical Research, Jewish General Hospital, 3755 Côte Ste. Catherine, Montreal, QC, H3T 1E2, Canada; 4 Department of Oncology, McGill University, Montreal, QC, Canada

## Abstract

In Genetic Analysis Workshop 18 data, we used a 3-stage approach to explore the benefits of pathway analysis in improving a model to predict 2 diastolic blood pressure phenotypes as a function of genetic variation. At stage 1, gene-based tests of association in family data of approximately 800 individuals found over 600 genes associated at *p*<0.05 for each phenotype. At stage 2, networks and enriched pathways were estimated with Cytoscape for genes from stage 1, separately for the 2 phenotypes, then examining network overlap. This overlap identified 4 enriched pathways, and 3 of these pathways appear to interact, and are likely candidates for playing a role in hypertension. At stage 3, using 157 maximally unrelated individuals, partial least squares regression was used to find associations between diastolic blood pressure and single-nucleotide polymorphisms in genes highlighted by the pathway analyses. However, we saw no improvement in the adjusted cross-validated *R*^2^. Although our pathway-motivated regressions did not improve prediction of diastolic blood pressure, merging gene networks did identify several plausible pathways for hypertension.

## Background

Pathway analysis of results from genetic association studies has become a very popular approach, and usually the goal is to gain a better understanding of which genes or groups of genes are related to the disease or trait being studied. However, "a better understanding" is not usually well defined. We chose to investigate whether pathway analysis could improve the *R*^2 ^value in a partial least squares (PLS) regression model predicting phenotype as a function of single-nucleotide polymorphisms (SNPs). Our hypothesis was that pathway analysis would highlight genes containing many SNPs with effects too small to be detected in univariate analyses, but that the PLS components (or latent variables) might benefit from such signals.

Analyses used a multistage approach to analyze associations with diastolic blood pressure (DBP) in the Genetic Analysis Workshop 18 (GAW18) data. At stage 1, we tested for association between sequence variation in each gene and DBP in the families. At stage 2, we built gene networks from the significant genes identified in stage 1, using Cytoscape [1-3] and identified enriched pathways. Stage 3 included the PLS regression models on unrelated individuals using different sets of SNPs motivated by stages 1 and 2.

## Methods

### Phenotype

We used 2 different DBP phenotypes: DBP at the first visit (DBP-1) and a measure of DBP change with age (DBP-C). In the GAW18 dataset, 795 individuals had both a DBP-1 measurement and genotype information; no covariates were used when analysing DBP-1. Slopes of DBP versus age were estimated from only 2 to 4 measurements in each of 855 individuals, and then slopes were adjusted for smoking and antihypertensive medication use, and categorized into 3 levels: none of the visits, some visits, or all visits. The resulting residuals formed our second phenotype, measuring whether an individual's blood pressure changes more or less than the average. There were 611 individuals with DBP-C and genotype information.

### Stage 1: ASKAT

Gene-based tests of association between the exome sequencing data and the DBP-1 and DBP-C phenotypes were performed using ASKAT [4], a method for quantitative phenotype analysis in families developed by our group. ASKAT fits a linear mixed model adjusting for the relationships by using the estimated kinship matrix. Kinship matrices were calculated for each odd-numbered chromosome using the genotype data in the chrx-geno.csv files. SNPs with minor allele frequency less than 0.01 were removed, and the kinship matrices were calculated using GenABEL v1.7-0 [5]. The average of all odd-numbered chromosome-specific kinship matrices was used in the gene-based ASKAT tests. Gene names were retrieved from hg19 build 37 (http://genome.ucsc.edu) [6], and we tested association between sequence-derived genetic variation (from the chrx-dose.csv files) in the exons of each gene and the DBP-1 and DBP-C phenotypes, using a total of 147,103 genetic variants. To optimize power to detect rare variants, the 201 genes containing more than 50 variants were divided into a series of non-overlapping windows with a maximum of 50 variants per window (based on empirical data from our group, not shown). For such genes, the minimum *p *value summarized the gene result.

### Stage 2: Pathway analysis

For DBP-1 and DBP-C, network analysis was performed using methods in Cytoscape 2.8.2 [7,8]. Networks were built using "Reactome FI" on genes with *p *value ≤0.05 from stage 1. The networks for DBP-1 and DBP-C were then compared and merged using "Advanced Network Merge-Intersection." Pathway analysis for genes in common was performed using the "Analyze Module Function."

### Stage 3: Regression analysis

PLS regression analysis was used for prediction modeling of our 2 phenotypes as a function of the number of minor alleles in SNPs identified by stages 1 and 2. PLS searches for multidimensional linear combinations of SNPs that explain the maximum variance direction of the phenotype, and can be thought of as constructing latent predictor variables. PLS models, using plsr version 2.3-0 of library pls [9], were fit to the 157 maximally unrelated individuals, using all variants from the chrx-dose.csv files, located in the genes identified by (a) *p *value ≤0.05 in gene-based analyses from stage 1, (b) stage 2 enriched pathways for DBP-1 or DBP-C with a false discovery rate (FDR) ≤0.05, or (c) stage 2 enriched pathways in the overlapping gene network. Tenfold cross-validation was used to choose the optimal number of PLS components, and we report the number of PLS components that gave the smallest adjusted cross-validated *R*^2^.

## Results

### Stage 1: ASKAT analysis

After analysis of 10,744 genes with ASKAT, 601 genes showed significant association with the DBP-1 phenotype (*p *value ≤0.05), whereas 694 genes were significant for the DBP-C phenotype. Among the 20 lowest *p *values for each phenotype, there were no genes in common (Table 1). The minimum *p *values were 6.31 × 10^−5 ^and 6.08 × 10^−6 ^for DBP-1 and DBP-C, respectively.

**Table 1:** Ten most significant genes from stage 1, ASKAT, with *p *values

DPB-1	*OSBPL3*	*GOBP1*	*HECW1*	*ZNF589*	*PARP1*
	6.31 × 10^−5^	7.21 × 10^−5^	0.000138	0.000299	0.000361
	*CCDC136*	*PLXDC1*	*NMUR2*	*C7orf69*	*LOC285954*
	0.000372	0.000389	0.000412	0.000494	0.000796

DBP-C	*C3orf24*	*TSR1*	*PEF1*	*NFP1P1*	*TESSP2*
	6.08 × 10^−6^	1.11 × 10^−5^	4.71 × 10^−5^	0.000109	0.000109

	*ISLR2*	*YWHAG*	*C15orf37*	*ZNF281*	*OR10H3*
	0.0002	0.000201	0.000251	0.000299	0.00036

### Stage 2: Pathway analysis

**Figure 1:**
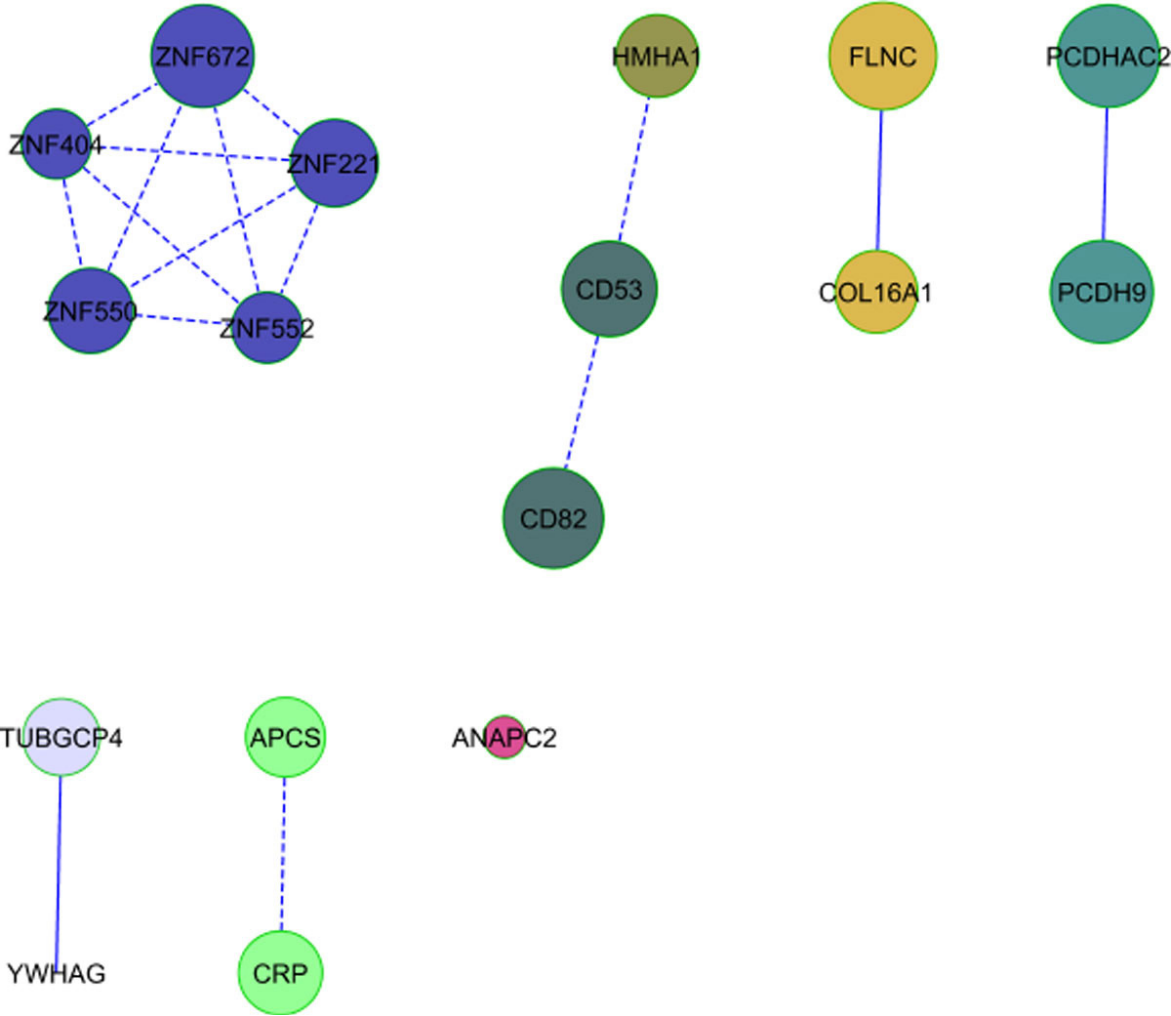
**Pathway analysis results**. Networks in common between the significant gene lists for DBP-1 and DBP-C. The size of the circle is inversely related to the gene's *p *value.

We found 84 enriched pathways (51 different genes) with FDR ≤0.05 for DBP-1, and 88 for DBP-C (59 different genes). There were 26 enriched pathways in common, but only the cadherin signalling pathway (P) and G2/M pathway (R) were part of the top 20 enriched pathways for both phenotypes. Merging the networks from the 2 phenotypes revealed some gene networks in common (Figure 1) containing 17 genes, and we estimated pathway enrichment in this set, finding 4 pathways that were enriched with FDR ≤0.05 (Table 2).

**Table 2:** Pathways enriched in overlapping networks of genes associated with DBP-1 and DBP-C

Pathway name	Gene name	*p *Value	FDR
Cadherin Signalling Pathway (P)	*PCDH9, PCDHAC2*	0.003	0.004
Wnt Signalling Pathway (P)	*PCDH9, PCDHAC2*	0.018	0.0425
Integrin Signalling Pathway (P)	*COL16A1, FLNC*	0.008	0.025
G2/M Transition (R)	*TUBGCP4, YWHAG*	0.001	0.005

### Stage 3-Regression analysis

Table 3 (fourth column) shows the optimal number of PLS components, identified by cross-validation, for the 2 phenotypes and for the 3 strategies for selecting SNPs. This approach identified either no components (linear combinations of SNPs) or 1 PLS component as providing the best fit to explain phenotype. However, even when 1 component was chosen as best, the improvement in error over the model with no components was minimal. To explore these results a bit differently, we measured the contribution per SNP for a 1-component PLS model, dividing the model *R^2 ^*by the number of SNPs in the PLS component that had nonzero regression coefficient (Table 3, last column). It can be seen that the pathway approaches improve this per-SNP contribution to *R^2^*. Also, the improvement in SNP importance across the 3 strategies is similar for either phenotype.

**Table 3:** Results from PLS regression analysis

SNP-selection strategy	Phenotype	# SNPs	C-V # PLS components*	*R*^2 ^with 1 PLS component†	Average *R*^2 ^per SNP‡
SNPs from genes with ASKAT *p *value ≤ 0.05	DBP-1	9414	1	0.418	7.1 × 10^−5^
	DBP-C	10,337	0	0.424	6.2 × 10^−5^

SNPs from genes in Cytoscape pathway with FDR ≤ 0.05	DBP-1	1239	0	0.333	4.4 × 10^−4^
	DBP-C	866	1	0.302	5.2 × 10^−4^

SNPs from genes in Cytoscape common network	DBP-1	242	0	0.228	1.6 × 10^−3^
	DBP-C	239	0	0.197	1.3 × 10^−3^

*The optimal number of PLS components, as determined by 10-fold cross-validation.

†*R^2 ^*values for a PLS model with 1 component.

‡The 1-component *R^2 ^*divided by the number of SNPs in the PLS component with nonzero regression coefficient, which are 5920, 6835, 759, 580, 143, and 150 SNPs, respectively.

## Discussion

Our pathway analysis was performed with Cytoscape, which is one among many possible pathway analysis tools. We also tried Gene Set Enrichment Analysis (GSEA) using the JAVA applet GSEA software v2.07 from the Broad Institute [10]. GSEA takes into account the gene rank, and tests whether a pathway is enriched by looking for an overrepresentation of genes at the top or bottom of a ranked list of *p *values. Using this method, no enriched pathway had FDR ≤0.05. However, a study comparing gene-set enrichment tests reported that GSEA was more consistent in finding enriched pathways [11].

Pathway analysis is often heralded as a solution for better understanding genetic effects, but how to best benefit from it is unclear. We selected a definition, improvement in *R*^2 ^of a multivariate (PLS) regression model, which is one of many possible definitions, and we explored this in GAW18 DBP data. We selected PLS regression in order to optimize construction of latent predictors, but the optimal number of PLS components identified through cross-validation was often zero for either phenotype and for any of the 3 gene and pathway selection strategies. We did not, in fact, find evidence for improved prediction of DBP using our strategy. However, PLS was not designed for categorical data and may not be the best choice for capturing associations between numerous SNPs and phenotype. When we forced a PLS fit with 1 component, the per-SNP contributions to *R^2 ^*from the overlapping genes were larger than when we used other approaches. It must be noted that although stage 1 used family data and stage 3 used unrelated individuals data, there is some overlap between these sets of individuals, and hence the per-SNP improvement in Table 3 may be partially explained by model over-fitting.

We chose to work with 2 DBP phenotypes, DBP at first visit, and a measure of DBP change with age. In most individuals, blood pressure increases with age. Our DBP-C phenotype, the residuals from a regression on age, measures each individual's sensitivity to the factors that lead to this general population trend of increasing DBP with age. We adjusted for medication use and smoking in a second model because with only 2 to 4 measurements per person, full longitudinal modeling was not possible. In this work, we have not adjusted for the variable precision of the slope estimates or age for DBP-1, and we recognize that this is a limitation.

Because our 2 phenotypes (DBP-1 and DBP-C) are closely related, we decided to focus our pathway analysis on gene networks present for both phenotypes. This strategy led to identification of 4 pathways significantly associated with DBP-1 and DBP-C. The first 3 pathways (see Table 2) interact together and play a role in pathogenesis of hypertension [12,13]. The G2/M transition pathway is known to be affected by leptin [14], a protein associated with hypertension [15].

Only odd-numbered chromosomes were included in GAW18, thus our identified pathways and networks will be incomplete. An analysis of the entire genome might validate our findings and provide additional significant associations. Furthermore, Cytoscape only assigned pathways to 120 genes out of the approximately 600 selected for either phenotype, thus providing further motivation to continue our analysis of a larger curated set of genes in pathways. Despite all the caveats in this work, it is interesting that our approach of examining overlapping pathways identified 3 pathways that are plausibly related to hypertension.

## Conclusions

For DBP or DBP changes in GAW18 data, we examined whether using pathway analysis results improved *R*^2 ^in multivariate regression models. Although we did not find evidence for improved model fits, 3 enriched pathways contained plausible hypertension-related genes.

## Competing interests

The authors declare that they have no competing interests.

## Authors' contributions

LD conducted statistical analyses and drafted the manuscript. KO designed ASKAT, and with VF, helped with the statistical analyses. CMTG supervised the statistical analyses and the manuscript. All authors read and approved the final manuscript.
